# Self-determination theory: its application to health behavior and complementarity with motivational interviewing

**DOI:** 10.1186/1479-5868-9-18

**Published:** 2012-03-02

**Authors:** Heather Patrick, Geoffrey C Williams

**Affiliations:** 1 Division of Cancer Control and Population Sciences National Cancer Institute 6130 Executive Boulevard Rockville, MD 20852-7335 USA; 2 Department of Medicine and of Clinical and Social Psychology University of Rochester 46 Prince Street Rochester, NY 14607 USA

## Abstract

Mounting evidence implicates health behaviors (e.g., nutrition, physical activity, tobacco abstinence) in various health outcomes. As the science of behavior change has emerged, increasing emphasis has been placed on the use of theory in developing and testing interventions. Self-determination theory (SDT)-a theoretical perspective-and motivational interviewing (MI)-a set of clinical techniques-have both been used in health behavior intervention contexts. Although developed for somewhat different purposes and in relatively different domains, there is a good deal of conceptual overlap between SDT and MI. Accordingly, SDT may offer the theoretical backing that historically has been missing from MI, and MI may offer SDT some specific direction with respect to particular clinical techniques that have not been fully borne out within the confines of health related applications of SDT. Research is needed to empirically test the overlap and distinctions between SDT and MI and to determine the extent to which these two perspectives can be combined or co-exist as somewhat distinct approaches.

## Self-Determination Theory: Its Application to Health Behavior and Complementarity with Motivational Interviewing

An impressive body of research has provided convincing evidence for the pivotal role of behavior in well-being, and morbidity and mortality, as well as health care costs [1]. Indeed, some estimates indicate that nearly 3/4 of all health care costs are attributable to chronic diseases resulting from health behaviors such as tobacco use and exposure, poor diet, and physical inactivity [2]. Other research has shown that adherence to five key lifestyle behaviors (eliminating tobacco exposure, body mass index (BMI) < 25, engaging in 30 minutes of physical activity or more per day, consuming alcohol in moderation, and eating a healthy diet) reduced coronary events by 62% over 16 years in a cohort of 42,000 US adult men. Further, men who adopted at least two of these behaviors had 27% lower risk for cardiovascular events compared to those who did not [3]. Lifestyle behaviors account for some 40% of mortality in industrialized countries and have been implicated in up to 2/3 of all cancers [4] as well as the onset and management of obesity, diabetes, cardiovascular disease, heart attacks, and stroke. Given the importance of health behaviors to well-being, health outcomes, and disease processes, developing a rigorous science of health behavior, its change and maintenance is critical to prolonging both length and quality of life.

In recent years, the science of health behavior change has increasingly emphasized theory- based approaches to intervention. The use of theory to inform and test interventions is important both for expanding basic science and for developing interventions that have real-world practical utility. From the perspective of basic science, theories must be tested in multiple domains and through multiple methods to refine and expand them appropriately. Further, the use of theory is important to applications in health behavior change and maintenance because theories often inform us on how interventions work by identifying underlying mechanisms, thus providing more proximal targets of intervention (i.e., mediators and moderators of intervention effects). Mediators may help to clarify the processes by which an intervention is efficacious and may be useful in circumstances when an intervention has either a direct or an indirect effect on the primary outcome. For example, an intervention may have no direct effect on a particular behavioral outcome but may indirectly improve the outcome via its effect on a psychosocial variable such as self-efficacy or motivation. Thus, interventionists may refine interventions to specifically target these intervening variables yielding more efficient interventions. In other circumstances, an intervention may directly impact a behavioral outcome, and mediators may elucidate the mechanisms through which an intervention functions and the sequence by which behavior change occurs. In this way, change in a mediator may be an important outcome in and of itself whereby interventionists and practitioners can gage whether an intervention is functioning in predicted ways prior to the assessment of the behavioral outcome at the end of an intervention (or other follow-up period). Moderators may help to clarify for whom and under what circumstances an intervention is efficacious (e.g., an intervention is particularly effective for a particular sociodemographic subgroup). Thus, interventions may target the populations for whom they are most efficacious and effective and/or be tailored to become more effective for other populations. Theory can also lead to paradigm shifts in how change and its maintenance are measured and how treatment outcomes are assessed. While a theory's parsimony, applicability to a range of behaviors and outcomes, and capacity to be refined and expanded are all important to basic science, good theories must also be practical. That is, theories must be rigorous not only from a scientific standpoint but also from a practical standpoint. To the extent that theories are consistent with clinical guidelines and tenets of clinical practice (e.g., medical professionalism, principles of biomedical ethics), they are better suited to not only scientific but also practical discourse.

The late 1970s and early 1980s saw the emergence of a key theory (i.e., self-determination theory; SDT) and clinical style (motivational interviewing; MI) that have been used to understand and intervene with health behavior. Although these efforts were spear-headed by two different groups and, to some extent, for two different purposes, today the parallels between SDT as a theory and MI as a style of clinical practice-as they apply to health behavior-are becoming increasingly clear [5-8]. These parallels have been further clarified as SDT researchers have developed efficacious clinical interventions based on SDT and MI techniques that facilitate health behavior change through change in the SDT mediators of autonomous self-regulation and perceived competence [e.g., [9-12]]. In addition, MI has moved toward a formal statement of theory in a recent publication [13]. Together, these events suggest that the synthesis of SDT (and its mediators) with MI techniques may be a potent combination that can contribute to the field of health behavior change. MI's movement toward a statement of theory also allows a closer comparison of common theoretical underpinnings between SDT and MI. We also offer discussion of some potential differences between MI and SDT not discussed previously. The purpose of this piece is to discuss self-determination theory and the more practical aspects of its application to health behavior in both research and clinical contexts and to further explore potential conceptual overlaps and distinctions between SDT and MI.

### Self-Determination Theory

Self-determination theory (SDT [14,15]) is a general theory of human motivation that emphasizes the extent to which behaviors are relatively autonomous (i.e., the extent to which behaviors originate from the self) versus relatively controlled (i.e., the extent to which behaviors are pressured or coerced by intrapsychic or interpersonal forces). SDT defines motivation as psychological energy directed at a particular goal. Many theories of human behavior account for the direction of behavior, but fail to account for how that behavior is energized [14]. SDT has thus emphasized the importance of motivational *quality *in addition to its *quantity*. It has also offered a particularly comprehensive approach to studying health behavior via its conceptualization and measurement of autonomy, perceived competence, relatedness to others, and its emphasis on the role of the social context in supporting or thwarting optimal motivation.

#### The Motivation Continuum

Traditionally, theories of motivation have made a distinction between intrinsic and extrinsic motivations. Intrinsic motivation is characterized by engaging in behaviors for their own sake, while extrinsic motivation is characterized by engaging in behaviors for some separable outcome, whether this comes in the form of tangible rewards, social acceptance, proving something to oneself, or maintaining consistency between one's values and one's behaviors. Given these definitions, many behaviors-particularly those relevant to health promotion (e.g., making dietary changes), disease prevention (e.g., screenings such as colonoscopy), and disease management (e.g., taking medications)-are likely extrinsic in nature [e.g., [16,17]]. However, not all extrinsic motivations are equivalent. Ryan and Connell [18] proposed a motivational continuum within SDT to better characterize the extent to which extrinsic motivations are relatively more or less internalized.

**Figure 1:**
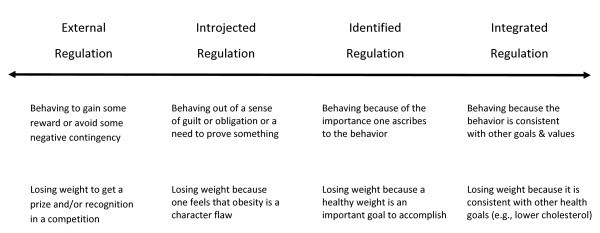
**The Extrinsic Motivation Continuum**.

SDT uses the term "internalization" to describe the process by which behaviors become relatively more autonomously regulated or valued over time. Autonomous self-regulation is particularly important for health behavior because the more autonomously-regulated an individual is toward a given behavior, the greater effort, engagement, persistence, and stability the individual is likely to evidence in that behavior [19]. According to SDT, the least internalized form of regulation is *external *and reflects engaging in behaviors to gain some reward or avoid some negative contingency. So, for example, someone may stop smoking because his surgeon will not perform needed coronary artery bypass surgery unless he stops smoking first, or because he wants the $800 his employer is offering to smokers for stopping. *Introjected *regulation involves engaging in behavior out of some sense of guilt or obligation or out of a need to prove something to oneself or others (i.e., enhance self-worth). Thus, a person may stop smoking because she would feel guilty about the emotional and financial turmoil her family would have to face if she were to have a prolonged illness and early death. The next most internalized form of regulation (i.e., the first level of autonomous regulation) is called *identified *in which case a person engages in a behavior because it is important to them. For example, someone may stop smoking because he personally believes it is an important goal to accomplish. Finally, the most internalized form of extrinsic motivation is *integrated*. Integrated regulations are motives for behaviors that are important to the person, and they are engaged because they are also consistent with one's other goals and values. So, someone may stop smoking because she values her health, and quitting smoking is consistent with her other goals in life (e.g., maintaining a regular exercise routine, living longer to enjoy her family). Figure 1 provides a visual representation of the continuum of extrinsic motivation. It is worth noting that, while described here as discrete, exclusive forms of motivation and self-regulation, it is quite common-particularly in health behavior-for different forms of regulation to coexist for the same behavior and to vacillate over time and across contexts. For example, someone may exercise because he values his health (identified regulation) but also because, as a health behavior researcher, he would feel guilty if he did not engage in the behavior he prescribes to patients, clients, or intervention study participants (introjected regulation).

#### Need Support: The Social Context and the Motivation Continuum

One of the defining features of SDT is its treatment of both the person (i.e., personality) and the situation (i.e., the social context) in motivated behavior. That is, at a personality level, individuals may orient to their surroundings in relatively more or less autonomous ways and thus their behaviors may be, on average, relatively more or less autonomously regulated. However, personality does not tell the full story. Indeed, the social context may support or thwart autonomous self-regulation and the process of internalization in any given domain. According to SDT, the extent to which one experiences need support from various contexts (e.g., doctor-patient interactions) is largely predictive of how autonomously-regulated one is likely to be for prescribed behaviors.

SDT has identified three psychological needs critical to supporting the process of internalization and the development of optimal motivation and personal well-being. The need for *autonomy *reflects the need to feel choiceful and volitional, as the originator of one's actions. *Competence *involves the need to feel capable of achieving desired outcomes, conceptually similar to self-efficacy in social cognitive theory. Finally, *relatedness *reflects the need to feel close to and understood by important others. When people experience the satisfaction of these needs in a given context, they are more likely to be autonomously self-regulated around the behaviors relevant to that context. Thus, to the extent that a patient feels his needs for autonomy, competence, and relatedness are supported in a discussion with his primary care doctor about, for example, modifying his diet to include more fruits and vegetables, the patient is likely to feel more autonomously self-regulated (i.e., more identified or integrated) around this recommended health behavior change.

Given the importance of need support in facilitating internalization, SDT has offered suggestions for specific behavioral strategies that may support one or more of these needs. For example, autonomy supportive behaviors include eliciting and acknowledging patients' perspectives and emotions before making recommendations; supporting patients' choices and initiatives; providing a rationale for advice given; providing a menu of effective (i.e., evidence-based) options for change; minimizing control and judgment; and exploring how relevant health behaviors relate to patients' aspirations in life. For example, a practitioner working with a patient on tobacco cessation may support the patient's autonomy by asking the patient to express what the patient thinks it would be like to deal with stressful situations without smoking (i.e., by eliciting the patient's perspectives and emotions). Competence support involves being positive that patients can succeed; reframing past failures as short successes; providing accurate effectance feedback in a non-judgmental manner; identifying barriers; skills building and problem solving; and developing a plan that is appropriately challenging to patients' skill and experience level. Competence support may be particularly relevant in the context of failure. For example, a patient may have gone two weeks without smoking but started smoking after a particularly stressful event at work. The practitioner could support the patient's need for competence by focusing on the accomplishment of being smoke-free for two weeks and discussing with the patient the important gains that were made in that (e.g., knowing that quitting for 2 weeks is possible; learning more about triggers for smoking, etc.). Both theoretical conjecture and some recent empirical evidence in applications of SDT to health suggest that authentic perceived competence does not emerge without the person feeling fully volitional (i.e., autonomously regulated [20]). Thus, supporting patient autonomy by ensuring that patients are fully volitional or willing to consider change is also relevant to supporting competence. Finally, relatedness support includes providing unconditional positive regard (particularly in the face of failure to achieve desired goals), being empathic with patients' concerns, and providing a consistently warm interpersonal environment. Thus, a practitioner may support a patient's need for relatedness by expressing understanding about how difficult making a behavior change like quitting smoking can be and reflecting the patient's concerns about failure. Support for all three needs requires that clinicians are actively engaged with their clients, and that they take a client-centered approach to the interaction. For example, eliciting and acknowledging the client's perspective starts with active listening and includes reflections (e.g., brief summaries of the thoughts, emotions, and plans the client has about the health issue being addressed). Typically perceived need support from health care practitioners has been measured with the Health Care Climate Questionnaire, (HCCQ [21-23]).

The concept of need support is one factor that makes SDT particularly amenable to health contexts as it is consistent with biomedical ethics [24], law and medicine [25], medical professionalism [26], and informed decision making [27,28]. Respect for patient autonomy is an integral part of all health care interventions. Biomedical ethics has elevated respect for autonomy to one of three highest- priority outcomes of all healthcare encounters, equivalent to those of enhancing patient welfare and improving social justice (e.g., eliminating discrimination). Thus, SDT's emphasis on supporting basic psychological needs, particularly individuals' need for autonomy, is consistent with these more general principles of patient care, making its practical utility in clinical and healthcare contexts paramount. These general principles of patient care also suggest that measuring autonomy and its change is important-if not essential-for translational research, as all clinical interventions are obligated to respect autonomy regardless of the theoretical frame it is based upon, or the outcome it is intended to change. Biomedical ethics establishes that respect for autonomy and autonomous self-regulation is an important health outcome in and of itself. This topic is addressed further by Vansteenkiste and Williams, this issue.

#### Aspirations

As described above, SDT has focused on the role of personality-level (i.e., motivational orientations) and domain-specific (i.e., self-regulation) forms of motivation, and has emphasized the importance of the social context in supporting or thwarting the process of internalization. SDT has also addressed the role of values and aspirations in goal pursuits [29,30]. Specifically, SDT has distinguished aspirations that are extrinsic from those that are intrinsic. Extrinsic aspirations reflect goals that are relatively external to the self and include wealth, fame, and image. Intrinsic aspirations reflect goals that are more internal to the self and include meaningful relationships, personal growth, community contributions, and, importantly, health. Research on aspirations within the SDT framework has focused largely on the extent to which people value intrinsic relative to extrinsic aspirations. When individuals place primary emphasis on extrinsic aspirations, they evidence lower levels of autonomy and relatedness as well as poorer physical and mental well-being and greater health risk behaviors. In contrast, placing stronger emphasis on intrinsic aspirations has been associated with a variety of positive outcomes including greater autonomy and vitality [29-32]. Recent interventions applying SDT to healthcare contexts have placed an emphasis on exploring patients' aspirations as a means of aligning the patient's broader life goals with goals for health behavior change. A newly-emerging body of research is also examining the internalization of aspirations over time (i.e., the extent to which individuals begin to shift focus from placing stronger emphasis on extrinsic aspirations to intrinsic aspirations [33]) and the role of aspirations in the context of health behavior change interventions. Indeed, recent analyses from the Smokers' Health Study (described below) indicated that an SDT-based intervention helped to sustain intrinsic aspirations at 12 months post-intervention. Further, intrinsic aspirations demonstrated both a mediating and a moderating effect on the intervention such that (a) an SDT-based intervention facilitated a maintained increase in the importance of intrinsic aspirations for health which in turn predicted better tobacco cessation outcomes at 18 months post-intervention; and (b) the SDT-based intervention was particularly effective in promoting long-term tobacco cessation amongst those who placed greater importance on health aspirations [34].

### From Basic Science to Application: SDT and Health

Unlike MI, which was developed in the context of health behavior change (i.e., problem drinking [35]), SDT was developed in the context of basic social science (i.e., theory development and testing [14,36,37]). Much of the early work on SDT focused on the undermining effects of rewards on intrinsic motivation [e.g., [38-40]], and the first applications of SDT were geared toward understanding these processes in education [e.g., [41-43]]. Over the past 10-15 years, a growing body of research has emerged testing the applicability of SDT to health contexts including the healthcare environment, health behavior change, and interventions. Together, the findings from these studies have demonstrated the role of need support and autonomous self-regulation in a variety of mental and physical health outcomes including depression, anxiety, somatization, quality of life, tobacco cessation, physical activity, weight loss, diabetes management, dental health, and medication adherence [20]. Below we summarize a few of these studies to highlight the breadth and depth of findings applying SDT to health.

Just as MI arose as a behavior therapy for problem drinking, one of SDT's first forays into health behavior application involved a study of individuals mandated to an 8-week alcohol treatment program [44]. Primary outcomes were attendance during the program and clinicians' ratings of patient involvement in treatment. Results revealed that individuals who had more autonomous self-regulation for alcohol treatment evidenced greater treatment attendance, program completion, and clinician-rated treatment involvement.

Williams and colleagues [22] studied individuals enrolled in a weight loss program for morbidly obese patients. The 26-week program involved a very low-calorie liquid diet for the first 13 weeks, with normal foods introduced gradually over the final 13 weeks of treatment. Treatment also included once weekly group sessions with 12-15 individuals to discuss feelings and challenges through the weight loss process, facilitate peer support, and provide techniques for self-monitoring diet, physical activity, and weight. Primary outcomes were program attendance and BMI change. Findings demonstrated that those with greater autonomous self-regulation for the weight loss program had better program attendance and greater reductions in BMI. Importantly, autonomous self-regulation for treatment predicted long-term BMI change more than one year after the end of treatment. Further, this study demonstrated a direct link between patients' perceptions of need support from treatment providers and (1) autonomous self-regulation for treatment mid-way through the intervention, (2) program attendance, and (3) BMI change (post-intervention and at long-term follow-up). Autonomous self- regulation for treatment mid-way through the intervention was shown to mediate the association between perceived need support and treatment outcomes, providing the first empirical evidence for the SDT model of health behavior change whereby the social context (i.e., need support) predicts motivation (i.e., autonomous self-regulation) which, in turn, predicts health behavior and/or health outcomes.

SDT has also been applied to the study of adherence to long-term and complex medication routines. In an observational study of 126 patients taking 1 of 30 different medications for more than two months (mean > 6 years), adherence over a 2-week period was strongly related to patients' autonomous self-regulation for taking that medication, as assessed at the beginning of the study. Importantly, patients' perceptions of need support from their healthcare providers also predicted autonomous self-regulation for medication-taking and for medication adherence [23]. Similar results were found in a study of 201 HIV+ patients on highly active anti-retroviral therapies (HAART). HAART medications regimens are particularly complex as they involve taking 3-4 medications several times each day. This study also included perceived competence for medication-taking and demonstrated an expanded model whereby perceived need support from health care providers predicted autonomous self-regulation for medication-taking which, in turn, predicted perceived competence for taking the medication. Perceived competence was a stronger and more proximal predictor of medication adherence [45]. This model was replicated in a study of medication use in a large closed health care system among more than 2,000 patients with diabetes, and the motivation variables prospectively predicted medication use, glycemic control and healthier cholesterol [46]. In other research, to the extent that patients with diabetes perceived their health-care provider to be need-supportive, they experienced greater autonomous self-regulation which, in turn, predicted perceived competence for both maintaining a healthy diet and exercising regularly. Perceived need support at Time 1 predicted lower blood glucose levels over 12 months both directly and indirectly through the links between perceived need support, autonomous self-regulation, and perceived competence [47]. Thus, autonomous self-regulation and perceived competence for the prescribed behavior seem to play an important role in behavior and health outcomes, and practitioners play an important role in facilitating these motivational variables.

Recently, researchers have begun developing interventions based on the tenets of SDT and the empirical support for the SDT process model of health behavior change from observational research. Here we describe a few of these interventions. Others are discussed elsewhere in this issue. Some of the earliest developments of SDT-based interventions involved tobacco cessation. In one study, 316 patients who smoked were recruited to have a discussion with their physician about their smoking behavior [21]. Physicians were randomly assigned to work with each patient in either a need-supportive or need- thwarting manner (i.e., randomization was at the level of patient) and to use the National Cancer Institute's 4As model for smoking cessation. The need-supportive condition was characterized by eliciting and acknowledging the patient's perspective, providing a rationale for advice given, and minimizing control, while avoiding judgment. The need-thwarting condition was characterized by the physician dominating the conversation, minimizing patient choice, and instructing the patient on what he or she should do, with no rationale or reflection. Patient involvement in the discussion was assessed by independent raters, based on audio tapes of the doctor-patient interaction. Patient smoking status was assessed at 6 months, 12 months, and 30 months post-discussion. Results revealed that observed physician interaction style indirectly predicted patient smoking status through its direct influence on patient involvement-a hallmark of autonomous self-regulation. This intervention is particularly important because it speaks directly to the practical utility of SDT as it was conducted in the real-world setting of a community-based physician group with physicians and their patients, with whom they had had ongoing relationships.

In the Smokers' Health Study, participants were 1,006 smokers who smoked at least five cigarettes per day and had smoked at least 100 cigarettes in their lifetime. Slightly more than half of the participants did not want to try to stop smoking at the time they enrolled in the study. Participants were randomly assigned to either an intensive treatment or community care control. Intensive treatment consisted of four contacts over six months. Practitioners were trained to interact with participants based on the Public Health Service Guideline intensive tobacco dependence treatment [48] in an SDT-consistent manner, which included: providing need support, including supporting the participant's decision about whether to stop or continue smoking; providing information about nicotine, tobacco dependence, and tips for successful quitting; exploring barriers and how smoking related to their values; using shared decision-making to develop a plan; problem-solving and skills-building; and access to pharmacotherapy. The community care condition consisted of provision of current pamphlets on stopping smoking and encouragement to discuss smoking with one's physician and was consistent with what was typically prescribed for tobacco cessation in the community at the time [49].

Results demonstrated support for the SDT process model whereby greater perceived need support from one's health care providers (including study practitioners) predicted greater increases in autonomous self-regulation and perceived competence for stopping smoking from baseline to the end of the intervention. Greater increases in autonomous self-regulation and perceived competence for stopping smoking predicted better tobacco abstinence 12 months after the end of the intervention, both in terms of 7-day point prevalence and prolonged abstinence. It is worth noting that autonomous self-regulation influenced tobacco abstinence indirectly through its impact on use of smoking cessation medications. This model was invariant across the intervention and community care groups, suggesting that internalization is at least, in part, a naturally-occurring process. However, it is important to note that those in the intervention group, compared to those in community care, evidenced greater perceived need support, greater changes in autonomous self-regulation and perceived competence for stopping smoking, greater medication usage, and higher abstinence rates. Thus, although the process of internalization appears to be a process that occurs naturally in the course of behavior change, this study demonstrated that the process can be accelerated through a need-supportive, SDT-based intervention [12]. Importantly, tobacco abstinence was maintained 24 months post-intervention more so for those in the intervention group compared to community care [50]. Thus, there is some initial evidence that SDT- based interventions not only facilitate health behavior change, but, importantly its maintenance. Further, change in autonomous self-regulation during treatment directly predicted 7-day abstinence 24 months post-intervention and indirectly predicted change in prolonged abstinence 24 months post- intervention. This suggests that the change in autonomy during treatment continued to motivate new efforts at abstinence well after the intervention was over.

SDT-based interventions have also been developed for dental behaviors and oral health [10]. Participants were 86 individuals in a dental clinic randomly assigned to either the SDT intervention or a usual care control group. All participants completed baseline questionnaires to assess autonomous self- regulation and perceived competence for dental care and were provided with a routine dental cleaning. One month following the dental cleaning, participants in the intervention group participated in a 60- minute informational session about dental health conducted by a dental hygienist. The informational session was designed to be consistent with the principles of SDT including acknowledging patient perspectives and feelings about dental health concerns, providing a rationale for dental prophylaxis, and providing choices and options for preventive behaviors that patients could choose to adopt. The dental hygienist also provided competence-support for intervention participants by demonstrating proper brushing and flossing techniques, allowing participants to practice these dental health behaviors, and conveying confidence in participants' ability to maintain these behaviors over time. Six months after the routine dental cleaning, all participants returned for an assessment of their oral health (plaque and gingivitis) and to complete follow-up questionnaires assessing autonomous self-regulation and perceived competence for dental care, self-reported dental behaviors, and attitudes and affect toward dental care. Compared to those in the usual care control, those in the SDT intervention group evidenced greater increases in autonomous self-regulation and perceived competence for dental care, decreases in plaque and gingivitis, better self-reported dental behaviors, and more positive attitudes and affect toward dental care. Importantly, further support for the SDT process model of health behavior change was provided by this study on dental health. Perceived need support of dental health providers predicted greater increases in autonomous self-regulation and perceived competence for dental care, which in turn predicted better dental health behaviors and outcomes (i.e., plaque, gingivitis).

In addition to these interventions developed for tobacco cessation and oral health, there has been a flurry of recent research activities involving SDT-based interventions for weight loss, physical activity, and dietary change. Although previous research has examined SDT variables in the context of traditional medical weight loss interventions [22], this recent research activity has used the tenets of SDT to inform the development of interventions for weight loss, physical activity, and diet. For example, in a study of patients in a community-based primary care practice, participants who worked with an SDT-trained physical activity counselor experienced greater need support in the health care climate which predicted greater increases in autonomous self-regulation for physical activity and, in turn, increases in perceived competence for physical activity. Both autonomous self-regulation and perceived competence for physical activity predicted greater increases in physical activity behavior [9]. In a one- year, SDT-based intensive behavioral intervention for weight loss among overweight and obese women, weight loss was greater for women in the intervention compared to the control at the end of the intervention and at 1 year post-intervention [11,51]. The intervention explicitly targeted increasing exercise autonomous self-regulation and intrinsic motivation, namely enjoyment of physical activity. The effect of the intervention on autonomous self-regulation was notable because it was large, it was sustained over one year, and it mediated the effect of the intervention on physical activity at 1 and 2 years [52]. Further evidence from this study has suggested a "motivational spill-over" whereby autonomous self-regulation for exercise predicted later autonomous self-regulation for healthy eating over one year [53]. Thus, facilitating autonomous self-regulation in one health domain may increase autonomous self-regulation in other, related domains. Additional details on each of these studies-and other related studies-are provided elsewhere in this issue.

Considered together, these randomized controlled trials demonstrate that SDT based interventions effect change in several health behaviors that are maintained after a free choice period (tobacco abstinence, physical activity, dental health, and weight loss). These tests of SDT interventions demonstrate mediation by key SDT constructs, thus linking SDT with these interventions' effect on important health behaviors through change in autonomous self-regulation and perceived competence. These studies conducted by several investigators in different countries (all western cultures) support a causal role of change in autonomous self-regulation, and perceived competence in the process of health behavior change.

### SDT and MI: Overlap and Distinctions

While SDT and MI have developed independently and have been utilized by relatively independent sets of researchers, recent attention has been given to the complementarity of these perspectives [e.g., [5,6]], including the MI-SDT Satellite Meeting held in Sintra, following the 2009 Annual Meeting of the International Society for Behavioral Nutrition and Physical Activity. It is worth noting that, in this section, we are considering the similarities and distinctions between SDT and MI through the lens of SDT researchers. Thus, it is possible (indeed, likely!) that those who view these two perspectives through the MI lens may see somewhat different similarities and distinctions [cf 7]. We hope that the points outlined below will facilitate further discussion, debate, and, perhaps most importantly, empirical investigation about how these two perspectives may complement and enhance each other and the science of health behavior more broadly.

Miller [54] has described MI as being based on concepts such as causal attributions, cognitive dissonance, and self-efficacy-all of which are grounded in social psychological theories and various social cognitive approaches. However, MI has been criticized for being largely atheoretical [55]. This lack of an organizing theoretical framework precludes explanations for how and why MI can be effective [56-58], although recent efforts have been made toward the development of an emergent theory of MI [13]. This is perhaps the most notable distinction between MI and SDT: SDT is a theory, while MI is a set of techniques (for further discussion of this distinction, see [8]). And although an advantage to SDT is that it offers a theoretical basis from which to understand the mechanisms through which SDT-based interventions are efficacious, a challenge to SDT researchers has been to translate theoretical concepts of need-supportive contexts into clinical techniques used in interventions. Thus, because of the consistency between MI techniques and SDT need support, many SDT-based interventions have been informed by MI techniques [e.g., [12,19]]. Importantly, SDT and MI have both drawn on Rogerian perspectives (e.g., unconditional positive regard, and patient centeredness [59,60]) and thus many of the underlying assumptions of both approaches are similar.

One of the areas in which much debate has ensued between SDT and MI researchers is around the area of directiveness. Although Miller and Rollnick [61] define MI as both client-centered and directive, MI is also very clear that attempts to directly persuade a client are ineffective in dealing with the client's ambivalence because such persuasive attempts inherently "take sides" in the ambivalence. In contrast, SDT has maintained, in the practice of healthcare interventions, that patient autonomy may be supported, in part, by making explicit recommendations about health and well-being (cf 12, 49). Further, in medical contexts in particular, explicit recommendations are often an expected component of interactions between practitioners and patients, and a practitioners' refusal to provide such direction-in addition to its potential for being unethical-does not support the patient's psychological needs. To illustrate, if a patient asks for a recommendation about treatment for a heart attack, the patient would likely feel a high level of control (e.g., thwarting of need for autonomy) and abandonment (thwarting of need for relatedness), for the doctor to insist the patient choose the treatment without a recommendation. Within SDT, recommendations must be given after eliciting and acknowledging client perspectives, non-coercively and in an autonomy-supportive way. When provided in this manner, the recommendation is more likely to be experienced by the patient as being informational, as opposed to coercive, and thus supports the patient in making the decision himself or herself (e.g., "I believe that stopping smoking is the best thing for your health, but only you can decide if you are going to smoke or not. The choice is ultimately yours, and I am here to support you in whatever decision you make."). More recent formulations of MI have allowed for medical practitioners to make recommendations when patients specifically ask for advice and have encouraged directiveness in the case of provoking change talk [13]. It is also possible that, as with the case of intrinsic motivation (described below), these two perspectives have defined "directive" in somewhat different ways.

Another distinction between MI and SDT is around the use of the term "intrinsic motivation." MI maintains that a primary goal of the techniques employed in MI interventions is to enhance intrinsic motivation [e.g., [61]]. However, SDT and other motivational theories [e.g., [62,63]] have defined intrinsic motivation as engaging in an activity for its own sake, because it is inherently enjoyable, satisfying or challenging. Given this definition, it seems likely that, rather than enhancing intrinsic motivation, MI techniques facilitate the process of internalization of extrinsic motivations (see [6] for a more detailed discussion of this point). This issue is largely one of semantics and may be one area in which SDT may serve to refine and enhance MI.

Despite these differences, there is actually a good deal of conceptual overlap and similarity between SDT and MI. Perhaps most noteworthy is that both SDT and MI start with the same basic assumption: That humans are naturally oriented toward growth, health and well-being. Additionally, both identify and work with-rather than attempt to combat-patient's ambivalence toward change. Further, MI techniques are at least partially consistent with SDT's notion of need support. Although traditionally, SDT has spoken primarily to the issue of autonomy support, the way in which perceptions of autonomy support have traditionally been measured (i.e., HCCQ [e.g., [17,21,22]]) and the nature of SDT-based interventions really address all three psychological needs. Indeed, perceived competence is facilitated by autonomous self-regulation, which arises out of need-supportive contexts [e.g., [20]]. Once individuals have a high willingness to act, they are more likely to learn new knowledge and apply new strategies that result in greater perceived competence. SDT predicts that perceived competence alone is not sufficient to motivate behavior; it must be accompanied by autonomy. This is in contradistinction to Social Cognitive Theory [64] which places nearly exclusive emphasis on self-efficacy.

As mentioned previously, MI techniques have informed some of the SDT interventions to date [e.g., [9,11,12]]. These SDT-based interventions are discussed in greater detail elsewhere in this issue [cf 64]. MI originally identified four key principles consistent with the practice of MI techniques: use of an empathic interpersonal style, development of discrepancy, rolling with resistance, and supporting self- efficacy for change [62,65]. More recent conceptualizations of MI applications to health care contexts have used somewhat different terminology, though the spirit of MI remains much the same [66]. Here we provide a brief overview of the current conceptualization of MI's four guiding principles (RULE = Resist the righting reflex, Understand and explore the patient's motivations, Listen to the patient empathically, Empower the patient) and three core communication skills (ask, listen, inform). We also discuss how these elements of MI are consistent with the support of psychological needs identified by SDT and thus, may support the process of internalization more broadly.

MI recognizes the natural tendency for those in the helping professions-particularly those in health care settings-to want to try to "fix" whatever is wrong with their patients or clients. However, MI also notes that resistance can arise when patients feel that their practitioner is trying to convince them of a particular course of action. This may be particularly pronounced in situations in which the individual feels ambivalent about change. Thus, it is critical that practitioners *resist the righting reflex *and instead allow clients to explore both sides of their ambivalence so that, in the end, the client is the one giving voice to reasons for change [66]. This guiding principle is similar to what SDT describes as minimizing control and remaining nonjudgmental. This may support clients' needs for both autonomy and relatedness by allowing patients the freedom to explore reasons for or against change (autonomy) in a non-judgmental context (relatedness).

Like SDT, from the perspective of MI, it is critical for patients to experience themselves as the originators of their actions toward behavior change. Thus, practitioners need to *understand and explore the patient's motivations*. This includes exploring how the patient views their current behavior and situation, concerns about change, and other goals and values [66]. This guiding principle of MI is consistent with SDT autonomy support, particularly eliciting and acknowledging client perspectives and emotions, supporting client initiative, and assessing values.

One of the defining features of MI is its emphasis on *listening to the patient empathically*. Thus, MI places importance on listening over informing on the part of practitioners, and an empathic interpersonal style, including an authentic interest in understanding the client [61,65,66]. According to MI, the client must feel personally accepted and valued before behavior change is possible. Listening to a patient empathically likely supports the client's need for relatedness and reflects that both MI and SDT emerged from the Rogerian school of thought, which promotes unconditional positive regard and patient centeredness as paramount to the therapeutic relationship [60]. Finally, the fourth guiding principle of MI-*empower the patient *- involves supporting self-efficacy for change. This technique likely primarily supports clients' need for competence by enhancing their confidence in being able to make progress toward positive change and to cope with challenges and barriers as they arise.

In addition to these four guiding principles, MI researchers have also articulated three core communication skills that provide practical utility to these principles. These communication skills include asking, listening, and informing. The purpose of *asking *is to elicit the client's perspective so that the practitioner understands where the patient is coming from and how the patient approaches the possibility of behavior change. *Listening *is an active process whereby the practitioner "checks in" with the client to ensure that he or she has an accurate understanding of client's perspective, motivations, and struggles through the process of behavior change. Finally, *informing *is the primary means by which practitioners convey knowledge to a client about their health condition, the behavior changes necessary to monitor or improve the health condition, and treatment options that may be available.

### Directions for the Future

Although SDT and MI originally began on two distinct paths, it seems clear from this special issue, the meeting in Sintra, and previous publications elsewhere [e.g., [5,6,67,68]] that there is now a critical mass of researchers engaged in dialogue about the similarities and distinctions between SDT and MI. However, it is important that this endeavor not stagnate with discussion and debate. Indeed, the next steps in this process of bringing together this theory and these clinical techniques must be borne out empirically. SDT has not yet identified the critical components for supporting psychological needs and facilitating autonomous self-regulation and perceived competence in health behavior interventions. MI techniques and their assessments may be useful additions to current SDT interventions in informing this empirical avenue.

Some MI interventions have included SDT measures [e.g., [69,70]], and this is an important step toward empirically testing the similarities between the two approaches. However, to date, results on whether MI interventions facilitate change in autonomous self-regulation, in particular, have been somewhat mixed, though some research has found that autonomous self-regulation mediates the association between MI intervention and treatment outcome [71]. Additional research is needed to identify which principles of MI operate on need support and the process of internalization. Further, current measures from SDT may need to be refined to better capture all three dimensions of perceived need support and to more accurately assess fluctuations in autonomous self-regulation and perceived competence in the initial stages of behavior change as well as behavior maintenance. In addition, assessment techniques, advanced analytic methods (growth curve analysis and latent trajectory modeling) and the use of internet technology and mobile devices in ecological momentary sampling may also improve researchers' ability to detect changes in motivation in response to provision of specific components of need support.

Finally, future research in which MI and SDT-based interventions are directly compared are needed to (1) more clearly elucidate the extent to which SDT can explain how and why MI interventions effect behavior, (2) identify aspects of SDT-based interventions that are similar to and distinct from MI (e.g., MI coding of SDT interventions and vice versa), (3) determine if MI interventions facilitate change in both autonomous self-regulation and perceived competence and support the need for relatedness, and (4) better clarify how both approaches can be used in concert to yield the most positive results. These empirical endeavors require not only a bridging of ideas but, perhaps more importantly, the convergence of a multi-conceptual team with representation from both SDT and MI camps to refine MI techniques, to improve SDT applications to health behavior, and to further expand our understanding of these approaches and how they serve to facilitate the initiation and maintenance of health behavior change.

Miller and Rose [13] recently published a statement on a theory for MI. Although SDT was not mentioned directly in that publication, SDT researchers may facilitate linking SDT to MI through empirical study of how eliciting change talk is experienced by clients (i.e., as relatively more or less need-supportive) and the extent to which change talk reflects a shift in patients' perceived locus of causality and/or change in autonomous self-regulation. From the SDT perspective, change talk is a reflection of the client or patient shifting from a voice of external locus of causality to internal -literally reflecting "real time" internalization. However, it is not clear that this is precisely how MI views change talk. Miller and Rose [13] and others have placed strong emphasis on practitioners actively promoting and eliciting change talk. This may be somewhat inconsistent with SDT. The aggressive push toward change talk may reflect an underlying assumption that the person is better off changing (when in fact this may not be their goal). Pushing change talk may be experienced as coercive and judgmental, and thus is not need-supportive. Self-determination theorists will also need to carefully consider whether MI's statement of theory is consistent with SDT. Particularly important for SDT theorists and researchers will be the resolution of issues such as MI's conceptualization of intrinsic motivation, the role of directiveness, and the issue of development of discrepancy. Though the latter is not explicitly listed in current conceptualizations of MI's guiding principles, the extent to which development of discrepancy is key to the execution of MI interventions may be important particularly with respect to whether this aspect of MI supports or thwarts need satisfaction.

## Conclusions

By bringing together the strengths of both approaches, we may be better equipped to develop efficacious interventions that yield positive results for health, health behavior, and well-being not only amongst the highly motivated participant pool willing to enroll in clinical trials but also among more general patient populations with whom practitioners interact on a daily basis. Only by facilitating the development of practical interventions with long-lasting effects will we succeed in improving length and quality of life through lifestyle change. The opportunity at hand may be to identify SDT and MI as complementary approaches. Collaborative efforts between complementary approaches will foster the development of a rigorous science of health behavior change that is equipped to tackle these issues in the real world of health care practice.

## Competing interests

The authors declare that they have no competing interests.

## Authors' contributions

HP and GWC discussed the format and scope of the manuscript. HP wrote the initial draft of the manuscript, and GWC contributed to the writing of the manuscript. All authors read and approved the final manuscript.

## References

[B1] Schroeder SA We can do better-Improving the health of the American people New England Journal of Medicine 2007 357 1221 1228 10.1056/NEJMsa073350 17881753

[B2] Woolf SH The power of prevention and what it requires Journal of the American Medical Association 2008 299 2437 2439 10.1001/jama.299.20.2437 18505953

[B3] Chiuve SE McCullough ML Sacks FM Rimm EB Healthy lifestyle factors in the primary prevention of coronary heart disease among men: Benefits among users and nonusers of lipid lowering and anti hypertensive medications Circulation 2006 114 2 160 167 10.1161/CIRCULATIONAHA.106.621417 16818808

[B4] Kushi LH Byers T Doyle C Bandera EV McCullough M Gansler T Andrews KS Thun MJ American Cancer Society American Cancer Society Guidelines on nutrition and physical activity for cancer prevention: Reducing the risk of cancer with healthy food choices and physical activity CA: A Cancer Journal for Clinicians 2006 56 254 281 10.3322/canjclin.56.5.254 17005596

[B5] Markland D Ryan RM Tobin VJ Rollnick S Motivational interviewing and self-determination theory Journal of Social and Clinical Psychology 2005 24 811 831 10.1521/jscp.2005.24.6.811

[B6] Vansteenkiste M Sheldon KM There's nothing more practical than a good theory: Integrating motivational interviewing and self-determination theory British Journal of Clinical Psychology 2006 45 63 82 10.1348/014466505X34192 16480567

[B7] Resnicow K McMaster F Motivational interviewing: Moving from why to how with autonomy support International Journal of Behavioral Nutrition and Physical Activity 2011 this issue 10.1186/1479-5868-9-19 PMC3330017 22385702

[B8] Vansteenkiste M & Williams GC Self-determination theory and motivational interviewing as examples of development from a meta-theory (top-down) vs. from clinical experience up (bottom-up): Implications for theory development, research and clinical practice and interventions International Journal of Behavioral Nutrition and Physical Activity 2011 this issue 10.1186/1479-5868-9-23 PMC3315422 22385828

[B9] Fortier MS Sweet SN O'Sullivan TL Williams GC A self-determination process model of physical activity adoption in the context of a randomized controlled trial Psychology of Sport and Exercise 2007 8 741 757 10.1016/j.psychsport.2006.10.006

[B10] Münster Halvari AE Halvari H Motivational predictors of change in oral health: An experimental test of self-determination theory Motivation and Emotion 2006 30 294 305 10.1007/s11031-006-9035-8

[B11] Silva MN Markland D Minderico CS Vieira PN Castro MM Coutinho SR Santos TC Matos MG Teixeira PJ A randomized controlled trial to evaluate self-determination theory for exercise adherence and weight control: rationale and intervention description BMC Public Health 2008 8 234 10.1186/1471-2458-8-234 18613959 PMC2483280

[B12] Williams GC McGregor H Sharp D Levesque CS Kouides RW Ryan RM Deci EL Testing a self- determination theory intervention for motivating tobacco cessation: Supporting autonomy and competence in a clinical trial Health Psychology 2006 25 91 101 16448302 10.1037/0278-6133.25.1.91

[B13] Miller WR Rose GS Toward a Theory of Motivational Interviewing American Psychologist 2009 64 27 537 10.1037/a0016830 PMC2759607 19739882

[B14] Deci EL Ryan RM Intrinsic motivation and self-determination in human behavior 1985 New York: Plenum Publishing Co.

[B15] Deci EL Ryan RM The 'what' and 'why' of goal pursuits: Human needs and the self-determination of behavior Psychological Inquiry 2000 11 227 268 10.1207/S15327965PLI1104_01

[B16] Ryan RM Psychological needs and the facilitation of integrative processes Journal of Personality 1995 63 397 427 10.1111/j.1467-6494.1995.tb00501.x 7562360

[B17] Williams GC Deci EL Ryan RM Suchman AL, Hinton-Walker P, Botelho R Building health-care partnerships by supporting autonomy: Promoting maintained behavior change and positive health outcomes Partnerships in healthcare: Transforming relational process 1998 Rochester, NY: University Of Rochester Press 67 87

[B18] Ryan RM Connell JP Perceived locus of causality and internalization: Examining reasons for acting in two domains Journal of Personality and Social Psychology 1989 57 749 761 2810024 10.1037//0022-3514.57.5.749

[B19] Ryan RM Deci EL Self-determination theory and the facilitation of intrinsic motivation, social development, and well-being American Psychologist 2000 55 68 78 11392867 10.1037//0003-066x.55.1.68

[B20] Ryan RM Patrick H Deci EL Williams GC Facilitating health behaviour change and its maintenance: Interventions based on self-determination theory The European Health Psychologist 2008 10 2 5

[B21] Williams GC Deci EL Activating patients for smoking cessation through physician autonomy support Medical Care 2001 39 813 823 10.1097/00005650-200108000-00007 11468500

[B22] Williams GC Grow VM Freedman ZR Ryan RM Deci EL Motivational predictors of weight loss and weight-loss maintenance Journal of Personality and Social Psychology 1996 70 115 126 8558405 10.1037//0022-3514.70.1.115

[B23] Williams GC Rodin GC Ryan RM Grolnick WS Deci EL Autonomous regulation and adherence to long-term medical regimens in adult outpatients Health Psychology 1998 17 269 276 9619477 10.1037//0278-6133.17.3.269

[B24] Beauchamp TL Childress JF Principles of biomedical ethics 2001 NewYork, NY: Oxford University Press

[B25] Schneider K Greenberg L, Watson J, Lietaer G Existential processes Handbook of experiential psychotherapy 1998 New York: Guilford 103 120

[B26] European Federation of Internal Medicine ABIM Foundation Medical Professionalism in the new millennium: A physician charter Annals of Internal Medicine 2002 136 243 246 11827500 10.7326/0003-4819-136-3-200202050-00012

[B27] Woolf SH Chan ECY Harris R Sheridan SL Braddock CH Kaplan RM Krist A O'Connor AM Tunis S Promoting informed choice: Transforming health care to dispense knowledge for decision making Annals of Internal Medicine 2005 143 4 293 300 16103473 10.7326/0003-4819-143-4-200508160-00010

[B28] Braddock CH Edwards KA Hasenberg NM Laidley TL Levinson W Informed decision making in outpatient practice: Time to get back to basics Journal of the American Medical Association 1999 282 24 2313 2320 10.1001/jama.282.24.2313 10612318

[B29] Kasser T Ryan RM A dark side of the american dream: Correlates of financial success as a central life aspiration Journal of Personality and Social Psychology 1995 65 410 422 8366427 10.1037//0022-3514.65.2.410

[B30] Kasser T Ryan RM Further examining the american dream: Differential correlates of intrinsic and extrinsic goals Personality and Social Psychology Bulletin 1996 22 280 287 10.1177/0146167296223006

[B31] Grouzet FM Kasser T Ahuvia A Dols JMF Kim Y Lau S Ryan RM Saunders S Schmuck P Sheldon KM The structure of goals across 15 cultures Journal of Personality and Social Psychology 2005 89 800 816 16351369 10.1037/0022-3514.89.5.800

[B32] Williams GC Cox EM Hedberg V Deci EL Extrinsic life goals and health risk behaviors in adolescents Journal of Applied Social Psychology 2000 30 1756 1771 10.1111/j.1559-1816.2000.tb02466.x

[B33] Weinstein N Przybylski AK Ryan RM Can nature make us more caring? effects of immersion in nature on intrinsic aspirations and generosity Personality and Social Psychology Bulletin 2009 35 1315 1329 10.1177/0146167209341649 19657048

[B34] Niemiec CP Ryan RM Deci EL Williams GC Aspiring to physical health: The role of aspirations for physical health in facilitating long-term tobacco abstinence Patient Education and Counseling 2009 74 250 257 10.1016/j.pec.2008.08.015 18838243 PMC2660169

[B35] Miller WR Taylor CA West JC Focused versus broad spectrum behavior therapy for problem drinkers Journal of Consulting and Clinical Psychology 1980 48 590 591 7410657 10.1037//0022-006x.48.5.590

[B36] Deci EL The psychology of self-determination 1980 Lexington, MA: D. C. Heath (Lexington Books)

[B37] Deci EL Koestner R Ryan RM The undermining effect is a reality after all: Extrinsic rewards, task interest, and self-determination Psychological Bulletin 1999 125 692 700 10.1037/0033-2909.125.6.627 10589297

[B38] Deci EL Effects of externally mediated rewards on intrinsic motivation Journal of Personality and Social Psychology 1971 18 105 115

[B39] Deci EL Intrinsic motivation, extrinsic reinforcement, and inequity Journal of Personality and Social Psychology 1972 22 113 120

[B40] Deci EL Ryan RM Berkowitz L The empirical exploration of intrinsic motivational processes Advances in experimental social psychology 1980 New York: Academic Press 39 80

[B41] Flink C Boggiano AK Barrett M Controlling teaching strategies: Undermining children's self- determination and performance Journal of Personality and Social Psychology 1990 59 5 916 924

[B42] Skinner EA Belmont MJ Motivation in the classroom: Reciprocal effects of teacher behavior and student engagement across the school year Journal of Educational Psychology 1993 85 571 581

[B43] Valas H Sovik N Variables affecting students' intrinsic motivation for school mathematics: Two empirical studies based on Deci and Ryan's theory of motivation Learning and Instruction 1994 3 281 298 10.1016/0959-4752(93)90020-Z

[B44] Ryan RM Plant RW O'Malley S Initial motivations for alcohol treatment: Relations with patient characteristics, treatment involvement and dropout Addictive Behaviors 1995 20 279 297 10.1016/0306-4603(94)00072-7 7653312

[B45] Kennedy S Goggin K Nollen N Adherence to HIV medications: Utility of the theory of self- determination Cognitive Therapy and Research 2004 28 5 611 628

[B46] Williams GC Patrick H Niemiec CP Williams LK Devine G Lafata JE Heisler M Tunceli K Pladevall M Reducing the health risks of diabetes: How self-determination theory may help improve medication adherence and quality of life Diabetes Educator 2009 35 484 492 10.1177/0145721709333856 19325022 PMC2831466

[B47] Williams GC Freedman ZR Deci EL Supporting autonomy to motivate glucose control in patients with diabetes Diabetes Care 1998 21 1644 1651 10.2337/diacare.21.10.1644 9773724

[B48] Fiore MC Bailey WC Cohen SJ Dorfman SF Goldstein MG Gritz ER Heyman RB Jaén CR Kottke TE Lando HA Mecklenburg RE Mullen PD Nett LM Robinson L Stitzer ML Tommasello AC Villejo L Wewers ME Treating Tobacco Use and Dependence 2000 Rockville, MD: U.S. Department of Health and Human Services. Public Health Service Clinical Practice Guideline

[B49] Williams GC McGregor H Sharp D Kouides RW Levesque CS Ryan RM Deci EL A self- determination multiple risk intervention trial to improve smokers' health Journal of General Internal Medicine 2006 21 1288 1294 10.1111/j.1525-1497.2006.00621.x 16995893 PMC1924739

[B50] Williams GC Niemiec CP Patrick H Ryan RM Deci EL The importance of supporting autonomy and perceived competence in facilitating long-term tobacco abstinence Annals of Behavioral Medicine 2009 37 3 315 10.1007/s12160-009-9090-y 19373517 PMC2819097

[B51] Teixeira PJ Silva MN Coutinho SR Palmeira AL Mata J Vieira PN Carraça EV Santos TC Sardinha LB Mediators of weight loss and weight loss maintenance in middle-aged women Obesity 2009 281 1 11 10.1038/oby.2009.281 19696752

[B52] Silva MN Markland D Vieira PN Coutinho SR Carraça EV Palmeira AL Minderico CS Matos MG Sardinha LB Teixeira PJ Helping Overweight Women Become More Active: Need Support and Motivational Regulations for Different Forms of Physical Activity Psychology of Sport and Exercise 2010 11 591 601 10.1016/j.psychsport.2010.06.011

[B53] Mata J Silva MN Vieira Pn N Carraca EV Andrade AM Coutinho SR Sardinha LB Teixeira PJ Motivational "spill-over" during weight control: Increased self-determination and exercise intrinsic motivation predict eating self-regulation Health Psychology 2009 28 709 716 19916639 10.1037/a0016764

[B54] Miller WR Motivational interviewing with problem drinkers Behavioral Psychotherapy 1983 11 147 172 10.1017/S0141347300006583

[B55] Draycott S Dabbs A Cognitive dissonance 1: An overview of the literature and its integration into theory and practice in clinical psychology British Journal of Clinical Psychology 1998 37 341 353 10.1111/j.2044-8260.1998.tb01390.x 9784888

[B56] Miller WR Motivational interviewing: III. On the ethics of motivational intervention Behavioural and Cognitive Psychotherapy 1994 22 111 123 10.1017/S1352465800011905

[B57] Miller WR Motivational interviewing: Research, practice, and puzzles Addictive Behaviors 1996 21 835 842 10.1016/0306-4603(96)00044-5 8904947

[B58] Miller WR Enhancing motivation for change in substance abuse treatment 1999 Rockville, MD: Center for Substance Abuse Treatment Treatment Improvement Protocol (TIP) Series, No. 35

[B59] Patterson TG Joseph S Development of a self-report measure of unconditional positive self- regard Psychol Psychotherapy 2007 79 557 70 10.1348/147608305x89414 17312871

[B60] Rogers C On Becoming a Person: A Therapist's View of Psychotherapy 1961 London: Constable

[B61] Miller WR Rollnick S Motivational Interviewing 2002 2 New York: The Guilford Press

[B62] Csikszentmihalyi M Finding flow: The psychology of engagement with everyday life 1997 Basic Books

[B63] Lepper MR Green D Nisbett R Undermining children's intrinsic interest with extrinsic rewards: A test of the overjustification hypothesis Journal of Personality and Social Psychology 1973 28 129 137

[B64] Bandura A Perceived self-efficacy in the exercise of personal agency Psychologist 1989 2 411 24

[B65] Miller WR Rollnick S Motivational interviewing: Preparing people to change addictive behavior 1991 New York: Guilford Press

[B66] Rollnick S Miller WR Butler CC Motivational Interviewing in Health Care: Helping Patients Change Behavior 2008 The Guilford Press. New York, New York

[B67] Foote J DeLuca A Magura S Warner A Grand A Rosenblum A Stahl S A group motivational treatment for chemical dependency Journal of Substance Abuse Treatment 1999 17 181 192 10.1016/S0740-5472(99)00003-3 10531624

[B68] Ginsburg JI Mann RE Rotgers F Weekes JR Miller WR, Rollnick S Motivational interviewing with criminal justice populations Motivational Interviewing 2002 New York: The Guilford Press 333 346

[B69] Resnicow K Jackson A Blissett D Results of the Health Body Healthy Spirit Trial Health Psychology 2005 24 339 348 16045368 10.1037/0278-6133.24.4.339

[B70] Rubak S Sandbaek A Lauritzen T Borch-Johnsen K Christensen B General practitioners trained in motivational interviewing can positively affect the attitude to behaviour change in people with type 2 diabetes. One year follow-up of an RCT, ADDITION Denmark Scandinavian Journal of Primary Health Care 2009 27 3 172 179 10.1080/02813430903072876 19565411 PMC3413190

[B71] Fuemmeler BF Mâsse LC Yaroch AL Resnicow K Campbell MK Carr C Wang T Williams A Psychosocial mediation of fruit and vegetable consumption in the body and soul effectiveness trial Health Psychology 2006 25 474 483 16846322 10.1037/0278-6133.25.4.474

